# Decreased FOXO1 Expression Is Correlated with Poor Prognosis in Myelodysplastic Syndromes

**DOI:** 10.3390/curroncol29100545

**Published:** 2022-09-25

**Authors:** Zheng Zhang, Nanfang Huang, Feng Xv, Sida Zhao, Juan Guo, Youshan Zhao, Chunkang Chang

**Affiliations:** Department of Hematology, Shanghai JiaoTong University Affiliated Sixth People’s Hospital, Shanghai 200233, China

**Keywords:** FOXO1, myelodysplastic syndromes, immunity, prognosis

## Abstract

Myelodysplastic syndrome is one of the main hematological malignancies that threaten the health of the elderly. However, biomarkers which predict the progression and prognosis of MDS are still controversial and puzzling. FOXO1 gene plays an important role in a variety of intracellular functions, including tumor suppression and cellular immune regulation. However, there is no research report on the correlation between FOXO1 and the clinical features of MDS including immune environment. In this study, we observed that FOXO1 expression is associated with neutrophil count, blasts, chromosome and different MDS scoring systems. FOXO1 expression is closely related to MDS cell immune polarization, and the increase expression of FOXO1 is significantly related to the amplification of immune cell polarization ratio. In addition, FOXO1 expression is associated with progression-free survival and overall survival in MDS patients. Moreover, in a multivariate model FOXO1 low-expression was an independent predictor of poor survival in MDS. In summary, FOXO1 may play a candidate tumor suppressor in MDS, and FOXO1 is a useful independent prognostic predictor in MDS, and it may provide a candidate target therapy in future.

## 1. Introduction

Myelodysplastic syndrome is one of the main hematological malignancies that threaten the health of the elderly. Some of them died from infection or bleeding caused by bone marrow failure, and the other died from disease progression to leukemia [1]. Due to age and treatment limitations, MDS patients with different risk stratifications adopt different treatment strategies, but the treatment effect is limited [2,3]. In recent years, some gene mutations have been used to monitor the prognosis and progression of MDS, and to detect the recurrence of the disease after demethylation therapy or chemotherapy [4,5]. However, these biomarkers are neither very sensitive nor particularly specific for predicting the progression and prognosis of MDS [6]. Therefore, further research is important to find new biomarkers and provide targeted therapy.

Forkhead box (FOX) family of transcription factors, named after the forkhead box domain (also known as the winged helix domain), are a family of 19 sub-families that share a highly conserved DNA-binding domain of approximately 110 amino acids. The FOXO gene family mainly consists of four members: FOXO1 (also known as FKHR), FOXO3 (also known as FKHRL1), FOXO4 (also known as AFX1) and FOXO6. Except that the expression of FOXO6 is restricted to the central nervous system, FOXO1, 3 and 4 are differentially expressed in different types of human tissues. Among them, the FOXO1 gene is located on chromosome 13q4, has widespread expression, and has been shown to be a representative member of the FOXO family with key regulatory activities in transcription [7]. Studies have shown that the FOXO1 gene plays an important role in a variety of intracellular functions, including autophagy, cell cycle inhibition, apoptosis, oxidative stress and the DNA damage response [8,9]. The regulation of cellular immune by FOXO1 gene has also been extensively identified, FOXO1 controls antitumor immune responses and the homeostasis as well as development of immune cells, including natural killer (NK) cells, dendritic cells, B cells, macrophages, and T cells. Furthermore, as a cancer suppressor, reviving the activity of FOXO1 favors the excretion of tumor-infiltrating activated regulatory T (Treg) cells from tumor tissues. In short, FOXO1 has broad effects on the immune system [10,11,12].

Many studies have reported that FOXO1 has anti-tumor effects on a variety of tumors, including hematological tumors, digestive system tumors, prostate tumors and breast tumors [13,14,15,16]. But a related paradox is that some studies have shown that FOXO1 plays completely opposite roles in different tumors [17,18]. We conducted a preliminary study on the role of FOXO1 gene in the pathogenesis of MDS. The results of the study seem to suggest that FOXO1 gene plays a tumor suppressor role in the pathogenesis of MDS [19]. However, there is no research report on the correlation between FOXO1 and the clinical features of MDS including immune environment. Therefore, this study aims to study the expression of FOXO1 in different stages of MDS, and to explore its clinicopathological, cellular immunity and prognostic relationship in MDS.

## 2. Materials and Methods

### 2.1. Patients

A total of sixty- eight MDS patients from our center between January 2016 and December 2018 was included in this study. The classification and prognostic risk scoring of MDS were performed according to the WHO criteria [20] and the IPSSR [21]. Survival duration was calculated from the date of diagnosis until 31 December 2019 when any remaining survivors were included. Progression-free survival (PFS) was defined as the time from the date of diagnosis to relapse or death as a result of any cause. Overall survival (OS) was measured from the date of diagnosis to death as a result of any cause. Disease relapse of MDS was judged according to the criteria of IWG in myelodysplasia [22]. Meanwhile, 10 normal or iron deficiency anemia patients served as normal controls, which exhibited no evidence of cancer and had normocellular bone marrow with no sign of haematologic disease. All of the subjects provided written informed consent to be included in the study, and all of the study procedures and informed consent forms were approved by the institutional review board of Shanghai JiaoTong University Affiliated Sixth People’s Hospital and were in accordance with the Declaration of Helsinki. OS was defined as the time from random assignment to death as a result of any cause. PFS was defined as the time from random assignment to local or distant relapse or death as a result of any cause.

### 2.2. Real-Time Quantitative PCR 

According to the manufacturer’s instructions, total RNA extraction from bone marrow mononuclear cells (BMNC) were performed with RNeazyMini Kit (Qiagen, Hilden, Germany). Employing the RevertAidTM First Strand cDNA Synthesis Kit (Fermentas, Burlington, Canada), cDNA was synthesized with random priming from 10 mL of total RNA, following the manufacturer’s instructions. The mRNA expression levels of FOXO1 were detected by real-time quantitative PCR. The reverse transcription reactions and quantitative PCR were performed as described previously [19] on the ABI 7500 Real-Time PCR System (Applied Biosystems, Foster City, CA). The housekeeping gene GAPDH was used to normalize mRNA levels. The following PCR primer sequences were used: FOXO1 forward: 5’-ACCTGGGTGTTGGGAGGGCA-3’, reverse: 5’- GGAGTGGATAGGCCACGGCG-3’. Each PCR reaction was repeated three times. 

### 2.3. Cell Culture and Sorting 

In order to verify the difference of FOXO1 gene expression in different cells of MDS, BMNC from 5mL fresh bone marrow samples of 3 matched MDS patients were isolated. After cultured at 37 °C and 5% CO_2_ saturated humidity, BMNC in logarithmic growth phase were taken for subsequent cell isolation. According to the manufacturer’s instructions, CD3+ and CD34+ cells were isolated using CD3 and CD34 immunomagnetic beads (Miltenyi Biotec, 130-050-101, 130-046-702) respectively. According to the manufacture’s protocol, for positive selection, cell pellet was resuspended in 300 μL MACS buffer (Miltenyi Biotec, 130-091-222) and 1 × 10^8^ total cells were incubated with 100 μL of FcR blocking buffer (Miltenyi Biotec, 130-100-453). CD3 or CD34 microbeads were incubated together with cell pellet for 30 min in the refrigerator (2–8 °C). Cells were added and washed by 10 mL of MACS buffer, then centrifuged at 300× *g* for 10 min. After discarding the supernatant and resuspend the cells at 500 μL buffer, and sort CD3 + or CD34 + cells by LS magnetic columns (Miltenyi biotec, 130-042-401). The sorted cells were detected by flow cytometry, and the positive expression rate reached more than 90%. BMNC of three MDS patients were sorted before and after disease progression for subsequent RT-PCR detection.

### 2.4. Subset and Polarization of T Lymphocytes

Similar to previous literature reports [23], the flow cytometry antibodies used in the following tests were purchased from BD Biosciences. BMMC was incubated with phorbol-12-myristate 13-acetate (PMA) and Ionomycin (Sigma, USA) for 4 h at 37 °C. The cells were incubated with flow cytometry antibodies against CD3-PerCP and CD8-APC (Becton Dickinson) for 15 min in dark. Then, the cells were treated with IntraPreP permeabilization Reagent B (Becton Dickinson) and were stained anti-human IFNγ-FITC and IL-4-PE (Becton Dickinson) for 15 min. After the cells were washed with cold PBS, an appropriate amount of 50ul PBS was added, flow cytometry was performed in FACS Calibur, and the results were analyzed by CellQuest software. Cell subsets are defined as follows: Th1 (CD8-INF-γ+), Th2 (CD8- IL-4+), Tc1 (CD8+ INF-γ+), and Tc2 (CD8+ IL-4+).

### 2.5. Immunohistochemistry (IHC)

The FOXO1 protein expression in human MDS tissues was detected using immunohistochemistry. After treatment, the bone marrow tissue was made into paraffin embedded sections with a thickness of 4 µ m, baked at 60 °C for 2 h, dewaxed with xylene, and hydrated with ethanol with different concentration gradients. After antigen retrieval, the specimens were treated with 3% hydrogen peroxide for 30 min to inhibit endogenous peroxidase activity, and then incubated with 1% goat serum albumin to block nonspecific binding. Tissue sections were incubated with anti-rabbit FOXO1 polyclonal antibody (1:100; CellSignal) at 4 °C overnight. After washing, the tissue sections were treated with anti-rabbit secondary antibody (sigma) at room temperature for development, and fully rinsed with distilled water. Then the slices were counterstained with 10% hematoxylin, differentiated with hydrochloric acid and alcohol, dehydrated and transparent with different concentrations of xylene, and finally sealed. The degree of immunostaining of formalin fixed paraffin embedded sections was evaluated by two pathologists. The staining index was determined by the proportion of positively stained cells and the intensity of staining in bone marrow tissue. The percentage of positive cells was scored as follows: sections with <10% positive cells were scored as 0; 10–50% positive cells were scored as 1; 50–75% positive cells were scored as 2; >75% positive cells were scored as 3 points. Staining intensity was graded into four grades: 0 for no staining; 1 for weak staining (light yellow); 2 for moderate staining (yellow brown); and 3 for strong staining (brown). The staining index (0–9) was calculated as the product of the proportion of positive cells multiplied by the staining intensity score.

### 2.6. Statistical Analysis

All of the statistical analyses were carried out with the statistical software package SPSS 21.0. The Kruskal−Wallis and Wilcox test were used to analyze the relationship between FOXO1 mRNA expression and clinicopathological characteristics. The Chi-square and Fisher’s exact test were used to analyze the relationship between FOXO1 mRNA expression levels and immunity characteristics. The expression of FOXO1 was divided into high expression group and low expression group according to the median value. Additionally, bivariate correlations were computed by Spearman’s rank correlation coefficients. Patient survival was determined by a Kaplan-Meier analysis, and the differences were counted by the log-rank test. Cox’s proportional hazards regression model was applied to the multivariate analysis. A *p* value of <0.05 in all of the analyses was considered statistically significant.

## 3. Results

### 3.1. Patient General Features

In total, 68 patients were enrolled in this study. The follow-up cutoff date was defined as the end of December 2019. The median age was 65 years old (range 35–85 years), and the male-to-female ratio was 43:25. The MDS subtypes included refractory anemia with excess blasts 1 (EB-1, n = 24), refractory anemia with excess blasts 2 (EB-2, n = 11) and other MDS subtypes (MDS with single lineage dysplasia, MDS with multilineage dysplasia, MDS with ring sideroblasts, MDS with isolated del5q, n = 33). According to the IPSS-R risk category, 1 patient scored ≤1.5, 15 patients scored >1.5~3, 19 patients scored >3~4.5, 22 patients scored >4.5~6, and 11 patients scored >6. Among the 68 patients, 50 had very good and good karyotypes, 13 had intermediate risk abnormal karyotypes and 5 had poor and very poor abnormal karyotypes (Table 1).

### 3.2. FOXO1 Was Differentially Expressed in MDS with Different Clinical Parameters

**Figure 1:**
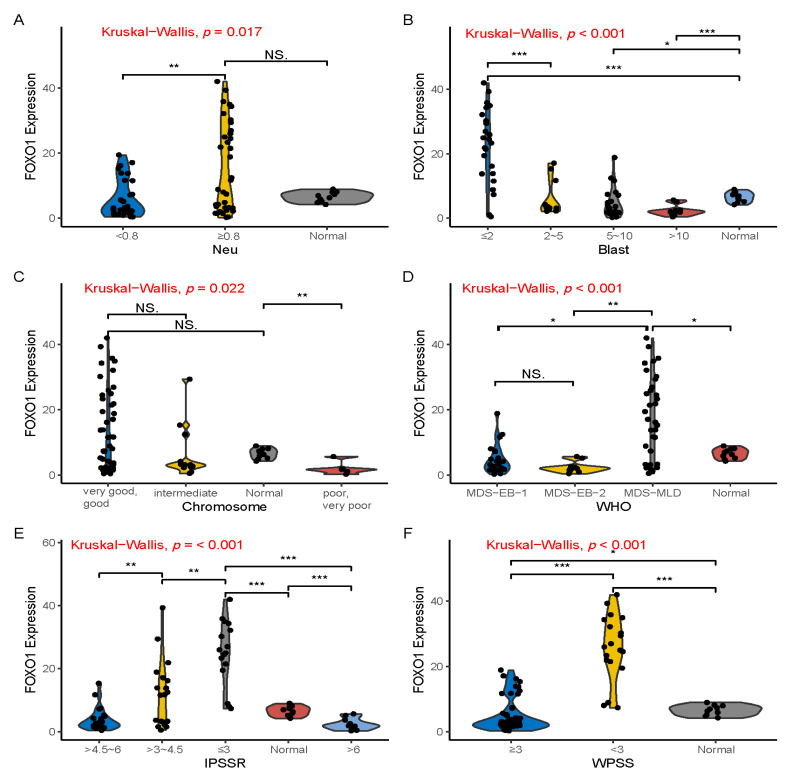
FOXO1 expression in MDS with different clinical features. (**A**) showed that the higher neutrophil count group had increased FOXO1 expression than lower neutrophil count group in MDS. (**B**) showed a significant decrease in FOXO1 expression with an increase in MDS bone marrow blasts, and the expression of FOXO1 in bone marrow blasts greater than 10% was significantly decreased in MDS patients. (**C**) showed that MDS patients with very good, good karyotypes had significantly higher FOXO1 expression than patients with poor, very poor karyotypes. (**D**) showed that FOXO1 expression was significantly higher in MDS-MLD patients than in MDS-EB1 and EB2 patients. (**E**,**F**) showed FOXO1 expression in lower-risk MDS was significantly higher than higher-risk MDS patients according to IPSSR and WPSS scoring systems. * means *p* < 0.05, ** means *p* < 0.01, *** means *p* < 0.001, NS means no significance. Abbreviations: Neu, neutrophil; WHO, World Health Organization; MDS-SLD, MDS-single lineage dysplasia; MDS-MLD, MDS-multilineage dysplasia; MDS-EB1, MDS-excess blasts-1; MDS-EB2, MDS-excess blasts-2; WPSS, WHO classification-based Prognostic Scoring System; IPSSR, Revised International Prognostic Scoring System.

To fully explore the significance of FOXO1 in MDS, we next divided into high and low FOXO1 expression groups according to the median value of FOXO1 mRNA, and several clinical parameters were evaluated. The data showed that there were significant differences in FOXO1 expression in different clinical characteristics of MDS, such as age, blasts, chromosome karyotype, WHO, IPSSR, WPSS and number of gene mutations (Table 2). In addition, we also analyzed the FOXO1 mRNA expression in different MDS clinical features, the data showed expression of FOXO1 were significantly different among clinical parameters such as neutrophil count, chromosome, WHO classification, IPSS-R and WPSS (Figure 1). Overall, FOXO1 expression was significantly decreased in MDS patients with an elevated proportion of bone marrow blast cells, poor karyotype, and high IPSSR and WPSS scores.

### 3.3. Relationship between FOXO1 Expression and Immunity Features in MDS Patients

**Figure 2:**
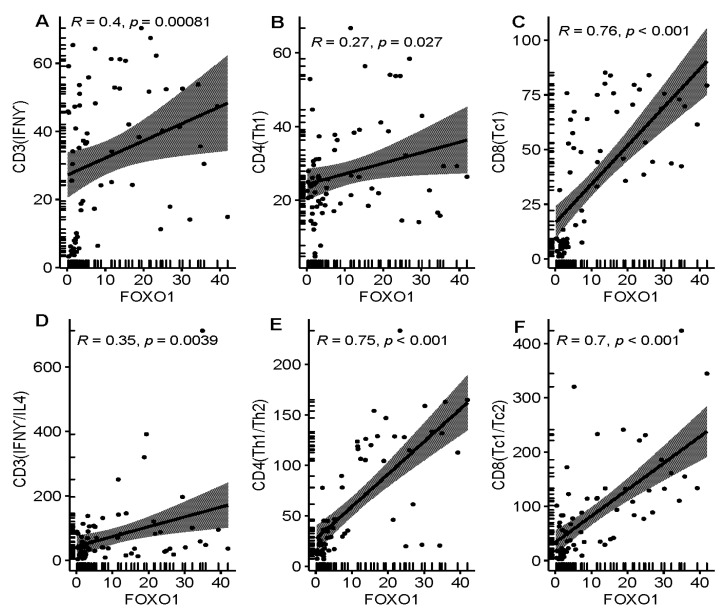
Association of FOXO1 expression with cellular immunity and polarization in MDS. (**A**) showed FOXO1 correlated weakly with CD3T (IFNƳ), (**B**) showed FOXO1 expression weakly correlated with CD4T (Th1), (**C**) manifested FOXO1 expression significantly correlated with CD8T (Tc1), (**D**) manifestd FOXO1 expression weakly correlated with CD3T polarization, (**E**) demonstrated FOXO1 expression significantly correlated with CD4T (Th1/Th2), and (**F**) demonstrated FOXO1 expression also significantly correlated with CD8T polarization.

**Figure 3:**
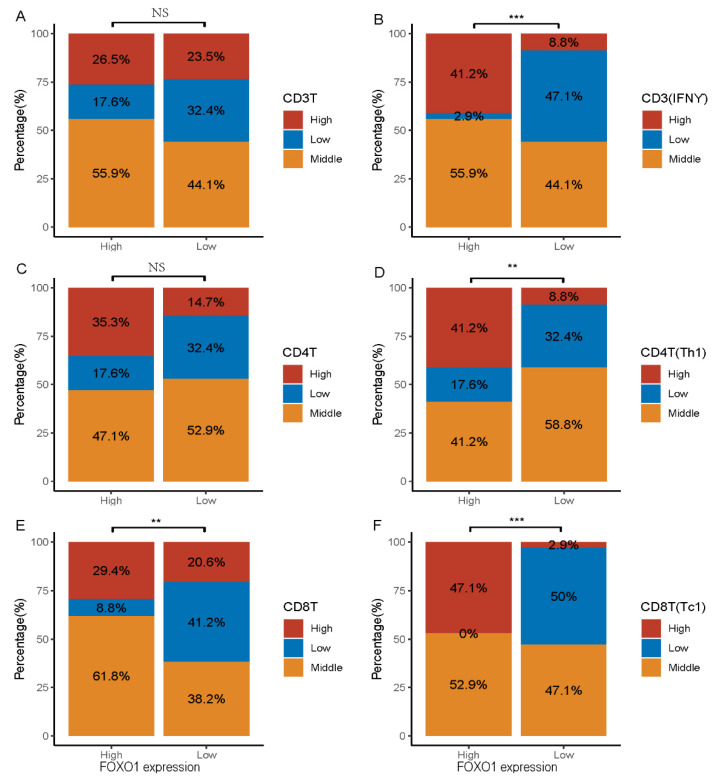
Effects of FOXO1 expression on immune cell distribution. FOXO1 expression higher than median value was defined as high expression, and FOXO1 expression less than median value was defined as low expression. Immune cell values higher than 75% range were defined as high expression, immune cell values between 75% to 25% range were defined as middle expression, and immune cell values below the 25% range were defined as low expression. We compared the percentage of low-, medium-, and high-expressing immune cells in the FOXO1 low- and high-expressing groups, respectively. (**A**) showed proportion of highly CD3T was high in MDS with high expression of FOXO1 relative to MDS patients with low expression of FOXO1, but not statistically significant. (**B**) displayed a significantly higher proportion of CD3T(IFNƳ) cells high expression in MDS with high expression of FOXO1 relative to MDS patients with low expression of FOXO1. (**C**) demonstrated MDS with high expression of FOXO1 had a higher proportion of high CD4T than those with low FOXO1 expression, but it was not statistically significant. (**D**) demonstrated the proportion of highly expressed CD4T(Th1) was significantly higher in the high FOXO1 expressing group than in the low FOXO1 expressing MDS patients. (**E**) illustrated the proportion of highly expressed CD8T was significantly higher in the high FOXO1 expressing group than in the low FOXO1 expressing MDS patients. (**F**) illustrated the proportion of highly expressed CD8T(Tc1) was significantly higher in the high FOXO1 expressing group than in the low FOXO1 expressing MDS patients. ** means *p* < 0.01, *** means *p* < 0.001. NS means no significance.

FOXO1 gene has the function of regulating immune cells. Therefore, we investigated the relationship between FOXO1 genes and immune cell subtypes. Spearman’s correlation analysis showed that high of FOXO1 expression was weakly correlated with CD3T(IFNƳ)(r = 0.4), Th1 (r = 0.27) and CD3 polarization(r = 0.35), and strongly correlated with Tc1(r = 0.76), CD4 polarization(r = 0.75) and CD8 polarization(r = 0.7) (Figure 2). We further analyzed the effects of different FOXO1 expression on immune cell distribution. Interestingly, the results showed that CD3T(IFNƳ), Th1, CD8T and Tc1 had higher percentage in high expression of FOXO1 as when compared to those in low expression (CD3T(IFNƳ) high 41.2 vs. 8.8%, *p* < 0.001, Th1high 41.2 vs. 8.8%, *p* = 0.008, CD8Thigh 29.4 vs. 20.6% *p* = 0.009, Tc1high 47.1 vs. 2.9%, *p* < 0.001) (Figure 3B,D–F). It is well known that cell polarization is one of the important characteristics of T cell function activation. These results suggest that FOXO1 play an important role in tumor immune surveillance of MDS.

### 3.4. Decreased Expression of FOXO1 Correlates with Disease Progression 

**Figure 4:**
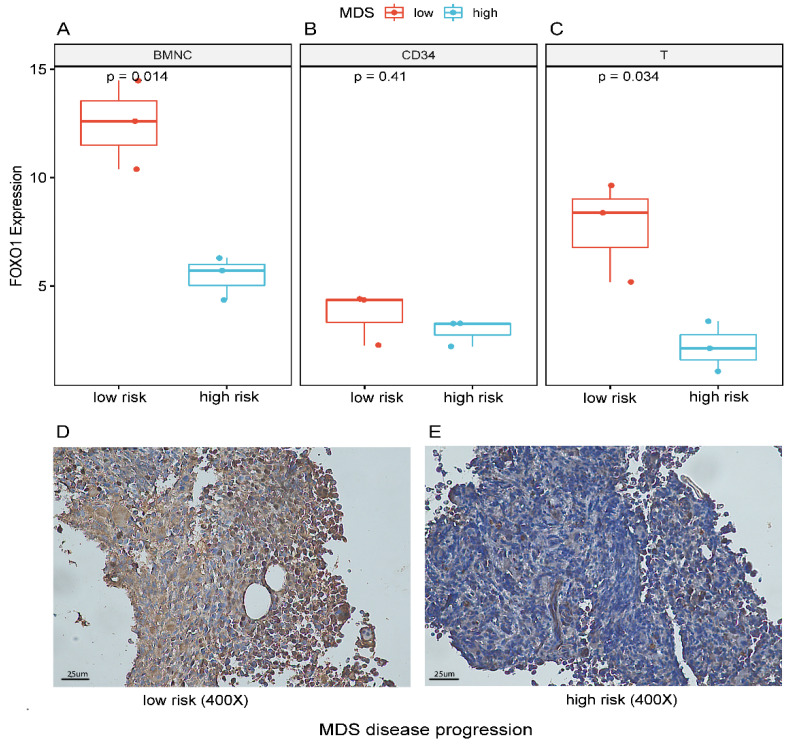
The expression FOXO1 gene and protein changed during MDS disease progression. During the progression of MDS from low risk to high risk, FOXO1 expression was detected in BMNC, CD34 and T cells, respectively. As MDS disease progresses, (**A**) showed a significant decrease in FOXO1 expression in BMNC, (**B**) demonstrated a slight but not statistically significant decrease in FOXO1 expression in CD34 cells, and (**C**) illustrated a significant decrease in FOXO1 expression in T cells. These results were further confirmed by immunohistochemical assays, with (**D**) demonstrating high FOXO1 protein expression in low-risk MDS and a significant decrease in FOXO1 protein expression in high-risk MDS (**E**) with disease progression. (scale bar: 25 μm)

We monitored FOXO1 gene expression in different cell components of 3 patients with MDS during disease progression. In general, FOXO1 gene expression in BMNC decreased after disease progression in these patients (Figure 4A). For CD34 cells, the expression of FOXO1 decreased compared with the previous one without statistical significance (Figure 4B). For T lymphocytes, the expression of FOXO1 decreased significantly, suggesting that FOXO1 gene plays a more important role in regulating T cell function (Figure 4C). To determine whether the FOXO1 protein expression was associated with disease progression in MDS, we stained bone marrow tissues of 3 MDS cases using IHC with a rabbit polyclonal antibody specific for FOXO1 during MDS progress. The FOXO1 staining index of 1 normal control was 3, and the staining index of 3 low-risk MDS were 9, 6, and 3, respectively. With the progression to high-risk MDS, the staining index decreased to 0, 3, and 0, respectively. The results showed FOXO1 protein was high in RCMD stage (Figure 4D). When the patient’s disease progressed to EBII stage, the expression of FOXO1 protein decrease dramatically, suggesting that FOXO1 gene plays an important role in cancer development of MDS (Figure 4E).

### 3.5. Low Expression of FOXO1 Correlates with Unfavorable Prognosis in MDS

**Figure 5:**
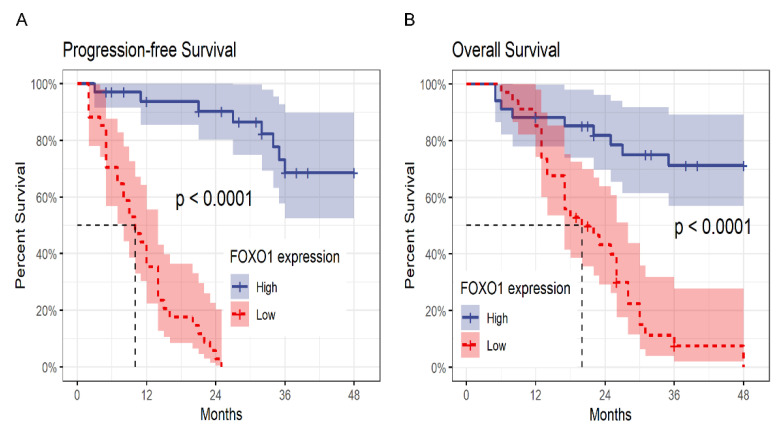
Progression-free survival and overall survival of myelodysplastic syndrome (MDS) patients according to FOXO1 expression. (**A**) Progression-free survival of 68 MDS patients was stratified by median of FOXO1 expression, MDS with high FOXO1 expression vs. MDS with low FOXO1 expression (*p* < 0.001). (**B**) Similarly, the overall survival of MDS patients with high FOXO1 expression was significantly longer than those with low FOXO1 expression (*p* < 0.001).

A survival analysis revealed that the cumulative PFS and OS rate of MDS patients decreased with reduction in FOXO1 expression. The median PFS and OS of patients with high FOXO1 expression did not reach, while patients with low expression of FOXO1 exhibited median PFS time of 9.5 months and median OS time of 20 months. Kaplan-Meier survival analysis exhibited that there was a statistically significance on PFS and OS between high and low expression of FOXO1 (*p* < 0.001) (Figure 5). 

In a univariate Cox analysis for PFS, FOXO1 expression, blast, chromosome, WHO, IPSSR and WPSS were significant prognostic factors. Moreover, in a multivariate Cox regression analysis found that low FOXO1 expression, >10% blasts and poor chromosome karyotype were indeed independent PFS factors of MDS (Table 3). Similarly, in the univariate Cox analysis of OS, FOXO1 expression, blast, chromosome, WHO, IPSSR and WPSS were also important factors affecting OS. In multivariate Cox regression analysis, it was found that low FOXO1 expression, poor karyotype and WPSS ≥ 3 were independent OS factors for MDS (Table 4). Taken together, all of these results suggest that FOXO1 expression was an indeed independent prognostic factor, and low FOXO1 expression may corelate with the poor prognosis of MDS.

## 4. Discussion

Studies have shown that FOXO1 plays an anti-tumor role in many solid tumors [15], such as breast cancer [24], prostate cancer [25], digestive system cancer [16,26] and other tumors [15]. It is confusing that FOXO1 seems to play the opposite role in solid tumors [26] and hematological tumors. Therefore, it has been controversial whether FOXO1 gene plays a role in tumor inhibition or promotion. At present, most scholars believed that FOXO1 played a tumor suppressor or tumor-promoting role completely depends on the tumor environment and disease stage in which it is located. Taking hematological tumors as an example, Köhrer found that in B-acute lymphoblastic leukemia, cell signaling activates the FOXO1 pathway and inhibits the occurrence and development of B-cell tumors, indicating that FOXO1 plays an anti-tumor role in B-ALL [27]. In addition, FOXO1 also inhibits the proliferation and growth of cancer cells in chronic myeloid leukemia (CML) and chronic lymphocytic leukemia (CLL) [28,29], thereby exerting antitumor effects in hematological tumors. However, other researchers found that FOXO1 promoted resistance of non-Hodgkin lymphomas to anti-CD20-based therapy and played a tumor-promoting role in FLT3-ITD+ AML through a complex mechanism, respectively [17,18]. Likewise, FOXO1 plays similarly paradoxical roles in different gastric cancer research settings [26]. However, in our study, we confirmed that, the expression of FOXO1 gene was disordered compared with the normal control, and there was a significant difference in the expression of FOXO1 gene in MDS bone marrow tissue. With the increase of the proportion of MDS blast cells, the expression of FOXO1 decreased significantly, suggesting that FOXO1 gene may play an anti-cancer role in MDS.

The difference of FOXO1 gene expression was closely related to the number of neutrophils, the proportion of bone marrow blast cells, chromosome karyotype, WHO type, IPSSR score and WPSS score of MDS. We found that FOXO1 gene expression decreased significantly in MDS-EB2 patients compared with MDS-MLD patients. Therefore, we speculate that FOXO1 gene plays a role of tumor suppressor gene in the occurrence and development of MDS disease. In order to confirm our conjecture, we monitored disease process of MDS and found that, not only the expression of FOXO1 gene decreased, but also the expression of functional FOXO1 protein decreased significantly by immunohistochemistry in the progress of the disease. The expression of FOXO1 gene in low-risk patients was significantly higher than that in high-risk MDS patients, indicating that low expression of FOXO1 is more likely to occur in advanced and high-risk MDS. Therefore, we consider that FOXO1 expression decreases with progression of MDS and can be used as a candidate molecular biological marker.

The formation and development of tumor was closely association with tumor immune microenvironment [30,31]. There were significant differences in the immune status of MDS patients in different disease stages. In patients with low-risk MDS, tumor antigen stimulated activation of immune system, and the activated immune system caused damage to its own hematopoietic stem/progenitor cells while killing MDS malignant clones, resulting in the reduction of whole blood cells that were difficult to recover. When patients progressed to a high-risk disease state, partly due to insufficient function of cytotoxic T cells, partly due to increased expression of immunosuppressive molecules such as PD-1, PD-L1 and Treg cells, which resulting in the escape of immune response and induction of immunological tolerance, promoted the progress of MDS to the high-risk stage [32]. Our results showed that the expression of FOXO1 was closely related to the number of CD8T (Tc1), CD4T polarization and CD8T polarization. In addition, we found that FOXO1 expression in T cells decreased significantly in higher risk stage when compared to those in CD34 cells. This was consistent with the findings of other previous studies [33,34,35], it was seemed that FOXO1 gene mainly regulated the expression of immune cells and its function. Interestingly, we further found that high expression of FOXO1 had higher ratio of CD3 (IFNƳ), Th1, CD8T and CD8T (Tc1) as when compared to those with low expression, suggesting that FOXO1 gene was closely related to expansion of number of immune cells and cell polarization in MDS. The strengthening of immune cell polarization was conducive to enhancing the immune effect. According to our results, it was speculated that low expression of FOXO1 causes functional decline of immune state, and immune surveillance for MDS malignant clone is significantly decreased, which may be an important reason for the progress of MDS disease. FOXO1, as a biomarker or predictor, is of great significance in predicting the immune status of MDS. Of course, more in vivo and in vitro experiments are needed to determine its role in MDS immune surveillance.

Even more critically, our results showed that low FOXO1 expression has an adverse effect on the overall survival (OS) of patients with MDS. Survival analysis showed that the cumulative OS rate of MDS patients decreased with the decrease of FOXO1 gene expression. In this study, the median overall survival of patients with low FOXO1 expression was only 20 months, while the median survival of patients with high FOXO1 expression was not reached. Multivariate Cox regression analysis showed that FOXO1 expression, chromosome karyotype and WPSS score were indeed independent prognostic factors of MDS, but the proportion of bone marrow blast cells, WHO classification and IPSSR score were no longer significant, suggesting that the decline of FOXO1 expression significantly affected the prognosis of MDS.

## 5. Conclusions

In conclusion, the results suggest that FOXO1 expression is an independent prognostic factor, and the low expression of FOXO1 may be related to the progression and poor prognosis of MDS. In the future, we need more in vivo and in vitro studies to clarify its role and molecular mechanism in the occurrence and development of MDS.

## Figures and Tables

**Table 1:** Clinicopathological characteristics in MDS.

Characteristic	n = 68 ^1^
Age(years)	
old (≥60)	48 (71%)
young (<60)	20 (29%)
Gender	
female	25 (37%)
male	43 (63%)
Neu (×10^9^/L)	
≥0.8	37 (54%)
<0.8	31 (46%)
Hb(g/L)	
<80	45 (66%)
80~<100	11 (16%)
≥100	12 (18%)
Plt (×10^9^/L)	
<50	37 (54%)
50~<100	14 (21%)
≥100	17 (25%)
Blast (%)	
≤2	24 (35%)
2~5	11 (16%)
5~10	22 (32%)
>10	11 (16%)
Chromosome *	
good, very good	50 (74%)
intermediate	13 (19%)
poor, very poor	5 (7.4%)
WHO (2016)	
SLD, MLD, RS-MLD, 5q-	33 (49%)
EB-1	24 (35%)
EB-2	11 (16%)
WPSS	
<3	30 (44%)
≥3	38 (56%)
IPSSR	
≤1.5	1 (1.5%)
>1.5~3	15 (22%)
>3~4.5	19 (28%)
>4.5~6	22 (32%)
>6	11 (16%)
Gene mutation	
<3	46 (71%)
≥3	19 (29%)
NA	3
Treatment	
cytokine	22 (32%)
immunosuppressive therapy	5 (8%)
lenalidomide	7 (10%)
hypomethylating agent	34 (50%)

^1^: n (%). *: Based on the IPSSR, cytogenetic risk categories are divided into very good, good, intermediate, poor, very poor. Abbreviations: Neu, neutrophil; Plt, platelet; WHO, World Health Organization; MDS-SLD, MDS-single lineage dysplasia; MDS-MLD, MDS-multilineage dysplasia; MDS-EB1, MDS-excess blasts-1; MDS-EB2, MDS-excess blasts-2; WPSS, WHO classification-based Prognostic Scoring System; IPSSR, Revised International Prognostic Scoring System.NA, not available.

**Table 2:** FOXO1 expression in association with standard clinicopathological variables in MDS.

Characteristic	High(N = 34) ^1^	Low(N = 34) ^1^	*p*-Value ^2^
Age			0.033
Old (≥60)	20 (59%)	28 (82%)	
Young (<60)	14 (41%)	6 (18%)	
Gender			0.8
Female	12 (35%)	13 (38%)	
Male	22 (65%)	21 (62%)	
Blast (%)			<0.001
≤2	22 (65%)	2 (5.9%)	
>2~<5	3 (8.8%)	8 (24%)	
5~10	7 (21%)	15 (44%)	
>10	2 (5.9%)	9 (26%)	
Chromosome			0.022
good	30 (88%)	20 (59%)	
inter	3 (8.8%)	10 (29%)	
poor	1 (2.9%)	4 (12%)	
WHO (2016)			<0.001
SLD, MLD, RS-MLD, 5q-	25 (74%)	8 (24%)	
EB-1	7 (21%)	17 (50%)	
EB-2	2 (5.9%)	9 (26%)	
IPSSR			<0.001
≤1.5	1 (2.9%)	0 (0%)	
>1.5~3	15 (44%)	0 (0%)	
>3~4.5	11 (32%)	8 (24%)	
>4.5~6	5 (15%)	17 (50%)	
>6	2 (5.9%)	9 (26%)	
WPSS			<0.001
<3	26 (76%)	4 (12%)	
≥3	8 (24%)	30 (88%)	
Gene mutation			0.047
<3	27 (82%)	19 (59%)	
≥3	6 (18%)	13 (41%)	
NA	1	2	

^1^: n (%). ^2^: Pearson’s Chi-squared test; Fisher’s exact test. Abbreviations: WHO, World Health Organization; MDS-SLD, MDS-single lineage dysplasia. MDS-MLD, MDS-multilineage dysplasia; MDS-EB1, MDS-excess blasts-1; MDS-EB2. MDS-excess blasts-2; WPSS, WHO classification-based Prognostic Scoring System. IPSSR, Revised International Prognostic Scoring System. NA, not available.

**Table 3:** Cox regression univariate and multivariate analyses of PFS factors in MDS.

Variable	Univariate Analysis		Multivariate Analysis	
	*p*	95% Confidence Interval	*p*	95% Confidence Interval
FOXO1				
Low expression	<0.001	9.66 (4.33–21.57)	**0.001**	5.24 (2.00–13.78)
Blast				
>2~5	0.006	3.85 (1.47–10.07)	0.519	1.79 (0.31–10.45)
>5~10	<0.001	7.16 (3.21–15.98)	0.180	3.88 (0.53–28.13)
>10	<0.001	16.16 (5.95–43.87)	**0.047**	1.55 (1.05–41.45]
Chromosome				
poor	<0.001	9.11 (3.08–26.99)	**0.002**	6.61 (1.99–21.97)
WHO				
EB-1	<0.001	4.30 (2.23–8.29)	0.75	0.714 (0.16–3.45)
EB-2	<0.001	9.91 (4.17–23.95)	NA	NA
IPSSR				
>3.5	<0.001	5.77 (2.52–13.19)	0.720	1.34 (0.27–6.65)
WPSS				
≥3	<0.001	5.07 (2.63–9.76)	0.923	1.06 (0.34–3.26)

Abbreviations: WHO, World Health Organization; IPSSR, Revised International Prognostic Scoring System; WPSS, WHO classification-based Prognostic Scoring System. NA, not available.

**Table 4:** Cox regression univariate and multivariate analyses of OS factors in MDS.

Variable	Univariate Analysis		Multivariate Analysis	
	*p*	95% Confidence Interval	*p*	95% Confidence Interval
FOXO1				
Low expression	<0.001	8.37 (3.53–19.85)	**0.029**	4.17 (1.16–15.06)
Blast				
>2~5	0.015	4.15 (1.31–13.15)	0.798	1.25 (0.22– 6.97)
>5~10	0.001	5.50 (1.97–15.37)	0.219	4.14 (0.43–39.85)
>10	<0.001	12.04 (4.13–35.12)	0.294	2.38 (0.47–12.03)
Chromosome				
intermediate	0.018	2.45 (1.17–5.15)	0.356	1.59 (0.59–4.28)
poor	0.004	4.50 (1.64–12.37)	**0.039**	3.29 (1.67–7.88)
WHO				
EB-1	0.013	2.70 (1.24–5.91)	0.296	0.36 (0.05–2.46)
EB-2	<0.001	6.62 (2.83–15.44)	NA	NA
IPSSR				
>3.5	<0.001	8.33 (2.53–27.44)	0.938	1.08 (0.15–8.06)
WPSS				
≥3	<0.001	8.05 (3.12–20.79)	**0.021**	4.26 (1.50–10.11)
				

Abbreviations: WHO, World Health Organization; IPSSR, Revised International Prognostic Scoring System; WPSS, WHO classification-based Prognostic Scoring System. NA, not available.

## Data Availability

The original contributions presented in the study are included in the Supplementary Materials, further inquiries can be directed to the corresponding author.

## Supplementary Materials

The following supporting information can be downloaded at: https://www.mdpi.com/article/10.3390/curroncol29100545/s1, Supplementary Materials File S1.
